# Subclinical tremor differentiation using long short-term memory networks

**DOI:** 10.1007/s13246-025-01526-0

**Published:** 2025-02-24

**Authors:** Gerard Ruchin Randil Nanayakkara, Ping Yi Chan

**Affiliations:** 1 Electrical and Robotics Engineering Department, School of Engineering, Monash University Malaysia, Jalan Lagoon Selatan, Bandar Sunway, 47500 Selangor Malaysia; 2 Centre for Net-Zero Technology, Monash University Malaysia, Jalan Lagoon Selatan, Bandar Sunway, 47500 Selangor Malaysia; 3 Medical Engineering & Technology Hub, School of Engineering, Monash University Malaysia, Jalan Lagoon Selatan, Bandar Sunway, 47500 Selangor Malaysia

**Keywords:** Subclinical tremor, Parkinson’s disease, Essential tremor, LSTM

## Abstract

Subclinical amplitudes complicate the differentiation between essential tremor (ET) and Parkinson’s disease (PD) tremor, which is uncertain even when the tremors are apparent. Despite their prevalence—up to 30% of PD cases exhibit subclinical tremors—these tremors remain inadequately studied. Therefore, this study explores the potential of artificial intelligence (AI) to address this differentiation uncertainty. Our objective is to develop a deep learning model that can differentiate among subclinical tremors due to PD, ET, and normal physiological tremors. Subclinical tremor data were obtained from inertial sensors placed on the hands and arms of 51 PD, 15 ET, and 58 normal subjects. The AI architecture used was designed using a long short-term memory network (LSTM) and was trained on the short-time Fourier transformed subclinical tremor data as the input features. The network was trained separately to differentiate firstly between PD and ET tremors and then between PD, ET, and physiological tremors and yielded accuracies of 95% and 93%, respectively. Comparative analysis with existing convolutional LSTM demonstrated the superior performance of our work. The proposed method has 30–50% better accuracies when classifying low amplitude tremors as compared to the reference method. Future enhancements aim to enhance model interpretability and validate on larger, more diverse datasets, including action tremors. The proposed work can potentially serve as a valuable tool for clinicians, aiding in the differentiation of subclinical tremors common in Parkinson’s disease, which in turn enhances diagnostic accuracy and informs treatment decisions.

## Introduction

Essential tremor (ET) and Parkinson’s disease (PD) are two of the most prevalent types of progressive neurological movement disorders in adults, affecting upwards of 10 million people worldwide [1]. Approximately nine in 1000 people are diagnosed with ET across all age groups, and up to 63 per 1000 over the age of 60 [2]. For PD, the prevalence is one or two individuals per 1000 people [3]. These disorders have a common cardinal symptom, i.e., tremor. While PD and ET have their own notable characteristics in terms of activation condition (rest or action tremor), frequency, and symmetricity (unilateral or bilateral), the great heterogeneity of characteristics makes them not entirely unique to each disorder. For instance, some PD patients have postural tremors that are phenomenologically similar to ET patients [4]. Thus, continuous improvement of clinical diagnostic criteria has been made over the past decades for better diagnostic accuracy [5].

As a matter of fact, these pathological tremors are difficult to analyse mathematically [6]. Efforts are made to differentiate between visible pathological tremors measured with various equipment, such as inertial sensors [7] and electromyography [8]. Statistical analysis of the tremor signals yields observations of the tremor characteristics that are still limited to aid in the differentiation in practice.

Thus, artificial intelligence (AI) has been proposed and used in tandem with sensors for better differentiation between tremors. Most of the previous work applies supervised machine learning such as random forest [9, 10], XGBoost [9], logistic regression, Gaussian process classifiers [10], k-nearest neighbors (KNN) [11–13], and own developed network [14] on accelerometer signals within the devices. Despite the known sensitivity to the sensor placement location when applying accelerometry compared to other inertial sensors that measure orientation, high classification performance was obtained in these previous studies. Recent AI-assisted tremor classification advancement focuses on the use of smart gadgets with better accessibility [10, 15], various input features [11], and novel architectures [14] in detecting visually obvious PD tremors from ET or healthy controls. Nevertheless, subclinical pathological tremors, which are more challenging in the differentiation, are not exclusively tested in previous studies.

Subclinical tremors are those with low amplitude and are exhibited by a much larger percentage of the population. These tremors are often physiological but can also be caused by neurological motor disorders. Although research shows that as much as 30% of PD patients display only subclinical rather than visible tremors [16], these tremors have not been widely studied.

In fact, a previous study investigates the characteristics of PD with low and high amplitudes, as well as of normal subjects. It is found that low amplitude tremors have less regular signal as quantified by approximate entropy and that the proportion of power in the least affected and the most affected limbs differ [17]. The difference in the characteristics of high and low amplitude suggests that PD tremors of varying severity deserve separate analysis [18].

To date, studies on the characteristics of subclinical PD and normal tremors are scarce. The earlier studies by Beuter et al. performed statistical analysis to identify the differences between visible and subclinical tremor due to PD [19]. At rest, differences were observed in terms of amplitude fluctuations, frequency dispersion, and proportional power in the 4–6 Hz frequency range. More studies also reported that other time- and frequency-varying properties related to the signals of linear displacement [19, 20] and joint motion [18] are significantly different in subclinical tremors of PD and normal subjects. Despite the statistical differences, the characteristics are highly overlapped. This highlights the difficulty of establishing baseline criteria to distinguish the individual case of subclinical tremor.

The use of AI in the classification of subclinical tremor has the potential to outperform conventional clinical and statistical analyses, which cannot reliably discern between the different types of subclinical tremor. To date, no prior studies have used AI in the differentiation of subclinical pathological tremors.

Therefore, this research project aims to apply deep learning, specifically long short-term memory (LSTM) networks, to differentiate among subclinical pathological tremors due to PD and ET and normal physiological tremors. In this work, we also explore the potential of using the short-time Fourier transform (STFT) that has three-dimensional information, i.e., time, frequency, and amplitude in differentiating tremors that are very difficult to differentiate by observation and manual signal analysis.

## Methodology

### Data acquisition and instrumentation

A clinical measurement study was carried out following approval from the Medical Research Ethics Committee, Secretariat of the National Institutes of Health, Malaysia [protocol no. NMRR-14–1694-21740 (IIR)] to measure and record tremors presented by several subjects with confirmed idiopathic PD and ET, and a healthy control group [21]. Doctors’ diagnoses of these pathological conditions were the gold standard based on which patients exhibiting tremors due to PD or ET were classified.

Tremor data were acquired from both healthy subjects and those diagnosed with PD and ET, using three microelectronic mechanical system attitude and heading reference systems (AHRSs) (model IG–500A, SBG System, Rueil–Malmaison, France) placed on the back of the patient’s hand, the distal end of the lower arm and the distal end of the upper arm [21]. Each sensor contained three gyroscopes, three accelerometers and temperature sensors, and output the 3D orientation, acceleration, angular rate, and magnetic field. Only the orientation data in the form of quaternions were processed, in order to avoid the effects of gravitational artefacts on the linear acceleration [22].

### Selection of subclinical data

The relative hand–arm motions, expressed in terms of the joint angle, were wrist abduction–adduction (WAA), wrist flexion–extension (WFE), elbow flexion–extension (EFE), and elbow pronation–supination (EPS) [21]. Following a study by Chan et al. [21], the average observational tremor rating of six medical doctors was related to the output from the tremor measurement system*.* Equation (1) relates the log mean square of the power for each of the joint angles measured to the doctors’ rating using a linear regression model.

1 $$Tremor \,rating=2.6496+0.3071*\mathit{log} {\theta }_{{WFE}_{MS}}+0.0731*\mathit{log}{\theta }_{{WAA}_{MS}}+0.1843 *\mathit{log}{\theta }_{E{PS}_{MS}}+ 0.0988\mathit{* }\mathit{log}{\theta }_{{EFE}_{MS}}$$

A rating of less than 0.5 was defined as subclinical tremor. For the purposes of this study, only resting tremor data were used for the neural networks, since Beuter et al. reported that in subclinical Parkinson’s tremor, resting tremor is more easily differentiable than postural tremor [19]. After identifying the subclinical tremors to be used and eliminating those with data corruption or unintentional movements, the final dataset consisted of subclinical tremor data from 58 normal subjects, 51 subjects with PD, and 15 with ET. Up to two readings were taken from each patient, contributing to a final dataset containing 122 physiological tremor readings, 83 subclinical PD tremor readings, and 30 subclinical essential tremor readings. The clinical characteristics of the subjects, including the stage of the disease and demographic data, are given in the “Results” section.

### Data processing

The orientation data were converted to the displacements of a reference point initially at (1, 0, 0) in the Cartesian domain. This reference point, *P*, represented mathematically as a vector in Eq. (2), underwent a rotation that was quantified in quaternion, $q$ (orientation data recorded by AHRS), as denoted in Eq. (3). The new point or position, $X$, after the rotation was calculated by multiplying the vector P by the vector q, followed by the multiplication of the resultant with the conjugate of q, denoted as q*, as elucidated in Eq. (4). The Eq. (5) shows the mathematical representation of q*. The displacement of the tremor with respect to time was then computed based on the difference between the position vector at an instance, t, and the original position vector at t_0_.

2 $${\varvec{P}} = \left(1, 0, 0\right)=1{\varvec{i}}+0{\varvec{j}}+0{\varvec{k}}$$

3 $$q = {q}_{0} +{q}_{1}{\varvec{i}} +{q}_{2}{\varvec{j}} +{q}_{3}{\varvec{k}}$$

4 $${\varvec{X}} = {q}^{*}{\varvec{P}}q$$

5 $${q}^{*} ={q}_{0}-{q}_{1}{\varvec{i}}-{q}_{2}{\varvec{j}}-{q}_{3}{\varvec{k}}$$

**Fig. 1:**
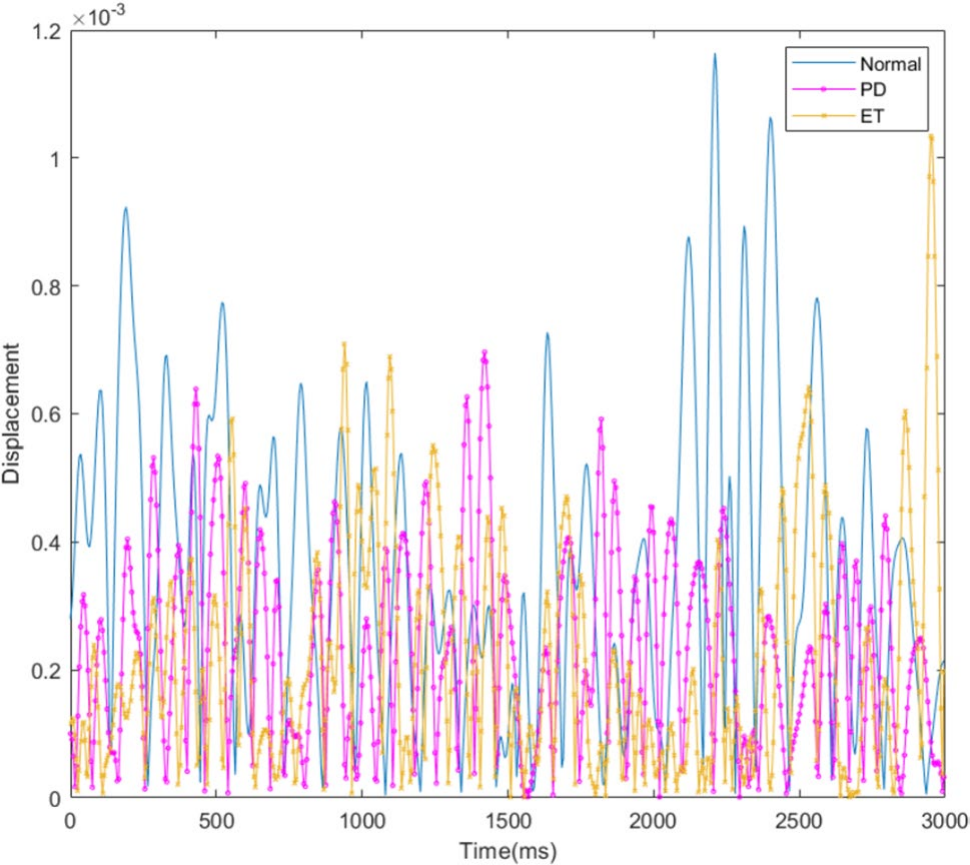
Displacement due to subclinical tremor

The typical frequency ranges over which subclinical tremors occur in PD and ET are around 3–9 Hz [23] and 4–14 Hz [24], respectively. Our data, which were sampled at a frequency of 100 Hz, were therefore adequate for this application, in accordance with Nyquist sampling theory. It was observed that in the data acquisition process using sensors, some data were lost during transmission. A piecewise cubic Hermite interpolation was therefore used to ensure that all datasets were recorded over the same time interval. A bandpass filter was applied to filter out any data outside the range 3–30 Hz. Since there were variations in the period of time over which data were collected for different patients, the data were processed and truncated such that each tremor measurement had a standard total duration of 13 s, the minimum measurement period found. Figure 1 shows the displacement of the hand sensor due to the different types of subclinical tremor over a period of 6 s. The signals of the three types of subclinical rest tremor have similar amplitude (dimensionless; the way to obtain the displacement is as mentioned in the previous paragraph) and pattern, which marks the level of difficulty in manual differentiation and the need for AI to overcome the traditional statistical and clinical analyses of subclinical tremors.

### Feature selection

Several feature selection methods were analysed, and a short-time Fourier transform (STFT) was applied to the tremor signal to allow it to be quantified and used as the input feature. The ‘stft’ function from the ‘Signal Processing’ toolbox in MATLAB2022a was applied to the time series data for displacement in the Cartesian frame. The STFT data were standardised to have a fixed resolution of 0.78 Hz and 320 ms for the frequency and time axes, respectively. The displacement plotted in the third dimension was a complex number that represented the magnitude and the phase. Since the time and frequency intervals were standardised, only the magnitude component of the displacement was fed into the classification algorithm, although the tremor information itself was intrinsically three-dimensional. The standardized frequency windows from the STFT data were then used as input features to the classifier. Since the data extracted from all three measurement units (located on the hand, upper arm, and lower arm) were the input, the feature set encompassed threefold the count of frequency windows. The data were then normalized in the batch normalization layer before feeding it into the neural network. It is important to note that the entire processing (pre-processing and AI network) was done in Python 3.11 except for the STFT and plotting, which were done in MATLAB.

### Classifying tremors with the LSTM network

**Fig. 2:**
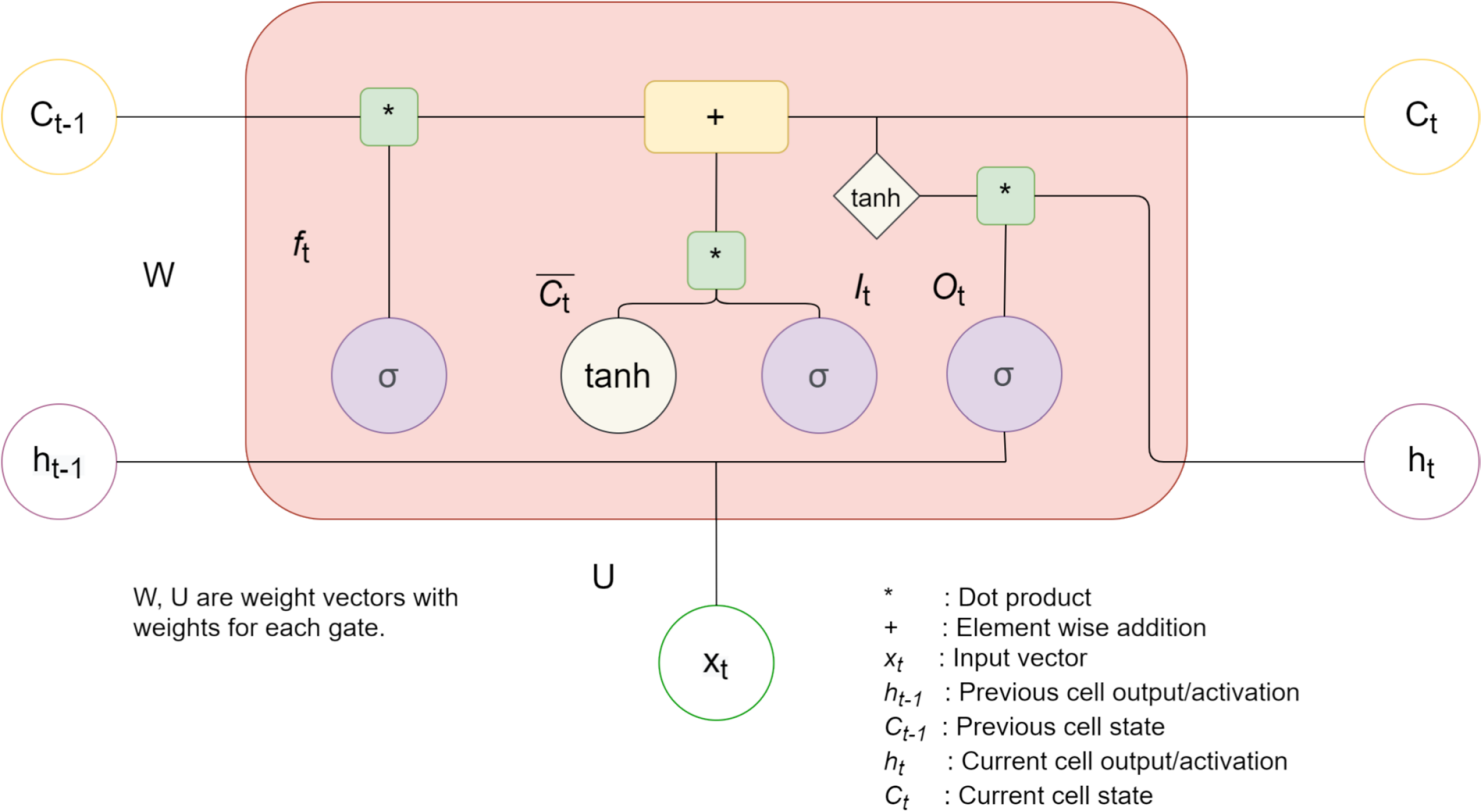
LSTM cell

The classifier algorithm in our work was implemented using an LSTM network. This is a type of recurrent network (RNN) that differs from traditional RNNs in that it is better at capturing long-term dependencies and applies a forgetting factor to determine the period of time and the degree to which past data affect decision-making [25]. Each LSTM cell consists of an input gate *I*_*t*_, a forgetting gate *f*_*t*_, an output gate *O*_*t*_, and a cell state *C*_*t*_ at each time instance, and it is computed using Eqs. (6) to (10). W represents the weights for each gate. A diagram of an LSTM cell is given in Fig. 2.

6 $${I}_{t}=\sigma ({X}_{t}*{U}_{i}+{h}_{t-1}*{W}_{i})$$

7 $${f}_{t}=\sigma \left({X}_{t}*{U}_{f}+{h}_{t-1}*{W}_{f}\right)$$

8 $${O}_{t}=\sigma ({X}_{t}*{U}_{o}+{h}_{t-1}*{W}_{o})$$

9 $${C}_{t}={f}_{t}*{C}_{t-1}+{I}_{t}*{\overline{C} }_{t}$$

10 $${\overline{C} }_{t}=tanh({X}_{t}*{U}_{c}+{h}_{t-1}*{W}_{c})$$

The activation state for each hidden unit h^n^ can be expressed as

11 $${h}^{n}={h}_{1}^{n}+{h}_{2}^{n}+{h}_{3}^{n}+\dots {h}_{t}^{n}$$

where h^n^ is the n^th^ hidden unit.

The Optuna framework, a hyperparameter optimization library for Python that employs Bayesian optimization and tree-structured Parzen estimators [26], was employed to iterate through, compare, and finalize the network architecture and hyperparameters. Since a full search of the hyperparameter space was not possible due to training time, the exploration focused on three critical hyperparameters known to significantly influence LSTM performance and generalization in time-series tasks [27, 28]

(i) numHiddenUnits ∈ {10, 300} with a step size of 10, controlling the capacity of the network to capture the temporal dependencies in tremor signals.
(ii) dropoutProb ∈ {${10}^{-6}$, 1.0}, to regularize the network by dropping a unit (along with connections) during training with a specified probability to prevent co-adaptation and therefore help generalization [29].
(iii) regFactor ∈ {${10}^{-5}$, 1.0}, the weight decay coefficient, which adds a penalty term, proportional to the square of the weights, to the loss function during training to mitigate overfitting by penalizing large weights.

The output of the LSTM layer was passed through a rectified linear activation (ReLU) layer and then connected to a fully connected layer, the specifications of which were varied and analysed using the Optuna framework. The number of layers of the fully connected layer and the number of hidden neurons per layer were varied in the ranges of ∈ {1, 3}, and ∈ {10, 300} with a step size of 10 respectively. The output of this fully connected layer was followed by a softmax layer and, finally, a classification layer. The final output of the network falls into one of three classes: normal (0), PD (1) or ET (2).

### LSTM training and validation

As described in section “Selection of subclinical data”, the subclinical tremor data available for training, validation, and testing consists of data from normal subjects (number of tremor data, n = 122), PD (n = 83), and ET patients (n = 30). Firstly, the data was split into two, with above 80% (82% for PD vs. ET and 87% for PD vs. ET vs. normal) being allocated for training and validation and the remaining allocated for testing. Then oversampling was employed to optimize the utilization of the collected data, by synthetically increasing the training dataset ensuring that any biases stemming from class imbalances were mitigated, as it was shown to outperform other methods in deep learning contexts [30]. Specifically, a modified version of time slicing [31] was applied to augment and oversample the minority class (ET) data to obtain a balanced training dataset. Time slicing which involves segmenting time series into windows, has been shown to improve deep learning model performance while preserving temporal dynamics [31, 32]. In this study, randomised overlapping time slices were obtained, and were zero-padded to vary the start time of these slices. Since local patterns and phase relationships are critical for distinguishing between subclinical tremors, this method allows for the effective expansion of the pathological tremor sample sets whilst faithfully representing the underlying tremor characteristics.

A categorical cross-entropy loss function was minimized to train the network. The Optuna framework was used again to optimize the batch size and the optimizer was used to train the proposed network. The batch size was varied from 16 to 128 by steps of 4, and the training performance of the ‘SGD’, and ‘Adam’ optimizers was observed. The batch size with the best performance was found to be 32, and the optimizer, Adam. During training, an early stopping algorithm was employed to stop training if the validation accuracy kept decreasing over a predefined time interval to ensure that the model did not overfit to the training data.

K-fold cross validation with k = 5, which samples according to the original distribution, was used to achieve a more robust validation. Prior to each full training loop, the data of each class was divided into five folds. One fold from each class served as validation data while the remaining four folds from each class were combined to be the training set. The folds used for validation and training were rotated, thereby ensuring that each data point had an opportunity to be both trained on and validated against, improving model generalization.

### Comparison of our method with existing methods on subclinical tremor data

No studies could be found that used AI-based classification systems for subclinical tremors. To assess the performance of our proposed methodology, we modified one of the recently published methodologies that used AI to differentiate visible tremor, in order to classify our subclinical tremor dataset collected from patients. This formed our benchmark algorithm and was adapted from Oktay and Kocer’s method [33], which was published in 2020. This was a convolutional LSTM network, i.e., it used a LSTM network connected to a convolutional neural network (CNN) to classify tremors as Parkinsonian or ET. It should be noted that their algorithm was originally applied to significant tremors rather than subclinical tremors. These authors used an LSTM network attached to the last layer of a CNN, which consisted of a convolution layer with 30 filters of length 20, a max-pooling layer, a second convolution layer with 30 filters of length 10, a max-pooling layer, and finally a fully connected layer. The architecture used a rectified linear activation function (ReLU) for activation of the convolution layers [33]. We replicated this approach using the same specifications and trained the resulting network on our subclinical tremor data. The performance characteristics and results from this method, when applied to subclinical tremors, were then compared with those of the method proposed in this paper.

**Fig. 3:**
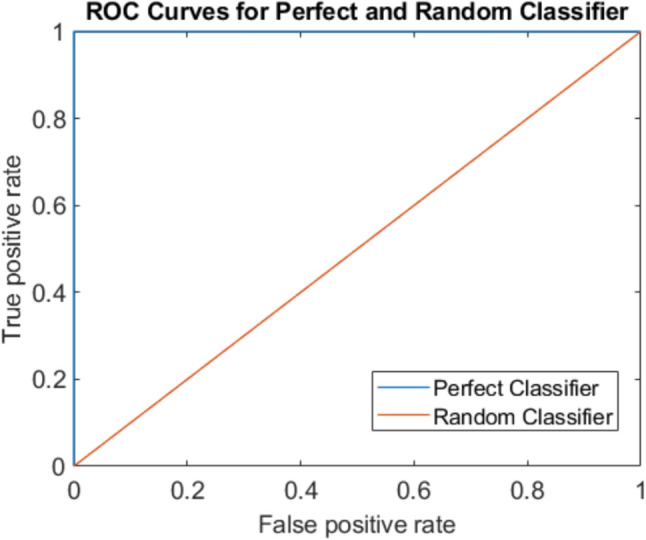
ROC curves for perfect and random classifiers

The receiver operating characteristic (ROC) curve can be used as a performance metric, and measures performance for classification problems at various threshold levels. A point on the ROC curve at any threshold value indicates the true positive rate and the false positive rate (FPR) at that threshold. The ROC curves for a perfect classifier and a random classifier are shown in Fig. 3. The area under the ROC curve (AUC) represents the overall degree of separability, meaning that it measures the capability of the model to distinguish between classes. An AUC value of close to one is ideal, indicating excellent classification, while an AUC of 0.5 means that there is no class separation and the model is not capable of differentiating between the classes. If the AUC is close to zero, then the model is inverting the classes, i.e. it is predicting negative classes as positive and vice versa.

## Results

The proposed classifier was used in two different ways to classify the types of subclinical tremors. The first experiment aimed to differentiate between subclinical tremors due to PD and ET. The performance metrics were then computed and compared with those of the convolutional LSTM classifier developed by Oktay and Kocer [33] for the same subclinical tremor test data.

As described in section “LSTM training and validation”, the network was trained and validated using k-fold validation (k = 5). The accuracy for each fold during training, validation, and testing is reported in Table 1. The results show that the training accuracy for each fold varies from 90.5 to 100%. Since each fold has 116 evenly balanced training samples, including oversampled data from the ET minority class as described in section “Comparison of our method with existing methods on subclinical tremor data”, the networks are able to classify 105–116 out of 116 samples accurately. The lower training accuracies in some folds are due to the precautions taken to minimize overfitting, such as early stopping and L2 regularization. These techniques seem to be effective as the validation and test accuracies are consistently high. The validation accuracies vary from 94.7 to 100%, with four folds having 100% accuracy on their respective validation sets. Each validation set included 18 or 19 samples, indicating that at worst, only one sample was misclassified. When tested on previously unencountered data, the network can classify the datasets with 90–100% accuracy, ensuring that the trained networks have generalized well and have not overfitted to specific data.

**Table 1:** Training, validation and testing statistics for classifying ET and PD tremors with the proposed classifier

	Fold 1	Fold 2	Fold 3	Fold 4	Fold 5	Mean accuracy
Training accuracy	0.905	0.948	0.964	0.977	1.000	0.959
Validation accuracy	0.947	1.000	1.000	1.000	1.000	0.989
Testing accuracy	1.000	0.950	0.950	0.950	0.900	0.950

Since accuracy is not sufficient as the sole performance metric for these classifiers, we computed the sensitivity (true positive rate), precision (positive predictive value), specificity (true negative rate), F1 score, FPR, negative predictive value (NPV) and AUC for both classifiers. These are summarised in Table 2, along with the classification accuracies.

**Table 2:** Performance metrics for PD vs ET classifiers on test data (PD as positive class)

	Accuracy	Sensitivity	Precision	Specificity	F1 Score	FPR	NPV	AUC
Proposed classifier	0.950	1.000	0.909	0.900	0.909	0.100	0.900	0.960
Convolutional LSTM [33]	0.650	0.600	0.667	0.700	0.632	0.300	0.636	0.740

For our classifier, an accuracy of 95% (refer to Table 2) is obtained based on the correct classification of 19 out of 20 tremor datasets. Of these, all 10 cases of PD tremors are identified accurately, and 9 out of 10 cases of tremors due to ET are correctly classified. The proposed classifier has a sensitivity of 1.000. In this case, sensitivity is defined as the ability to correctly classify PD tremors and was calculated by computing the number of true PD classifications divided by the actual total number of PD classifications. The precision (also known as the positive predictive value) was calculated by computing the number of true PD positives divided by the total predicted PD cases, including both correctly classified PD tremors and ET tremors classified as PD tremors. The classifier has a precision of 0.954, which indicates a good level of performance. The specificity is a measure of the proportion of ET tremors that are correctly identified and is calculated as the number of true ET classifications divided by the actual number of ET classifications. The specificity value of 0.900 achieved by the classifier shows that it can accurately classify ET tremors as well. This, along with the high sensitivity, implies that the system is not biased toward one class. The F1 score, which is the harmonic mean of the precision and recall, of 0.909 indicates near-perfect and unbiased performance. The false positive rate is defined as the number of false PD classifications divided by the total number of expected ET tremors. One ET case is incorrectly classified as PD out of a total of 10 ET cases, resulting in a false positive rate (FPR) of 0.100, which is sufficiently low to be considered satisfactory.

From Table 2, it can be inferred that the proposed classifier outperforms the convolutional LSTM method for every performance metric considered. An accuracy of 95% is achieved, as compared to 65% using the convolution LSTM model. The classifier far outperforms the convolutional LSTM method in terms of sensitivity, with a score of 100% versus 60%. Our method also scores better than the convolutional LSTM method across all metrics with better precision, specificity, F1 score, and NPV while maintaining a lower FPR.

**Fig. 4:**
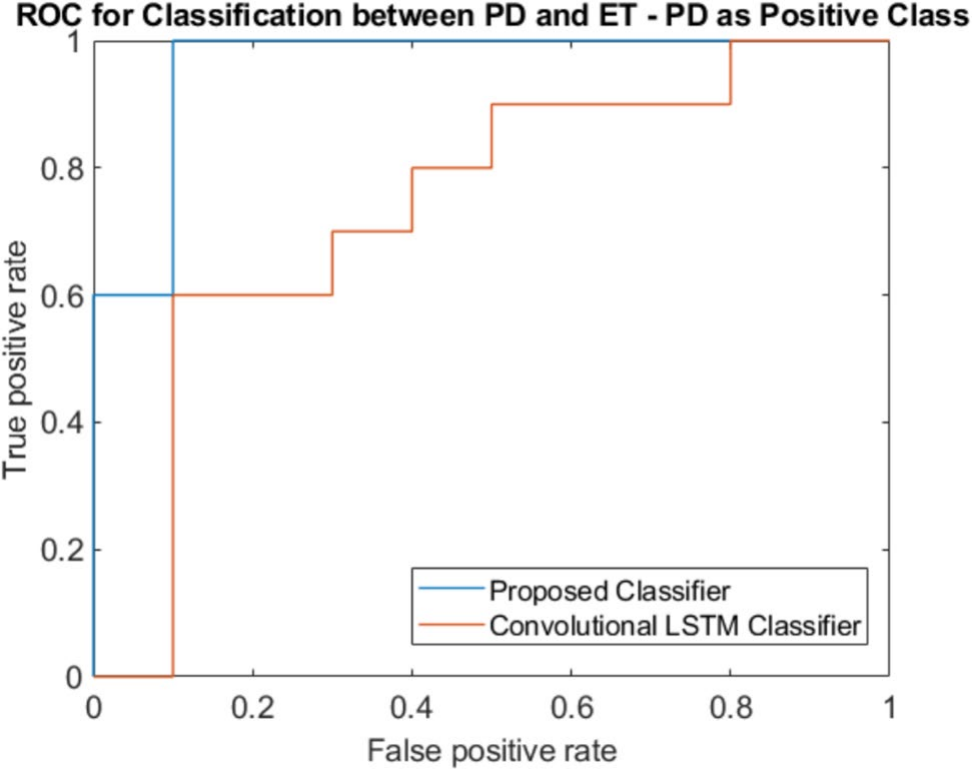
ROC curve for classifying between PD and ET

The ROC was calculated using the ‘perfcurve’ function from the ‘Statistics and Machine Learning Toolbox’ within MATLAB 2022a. The ROC curves for both our classifier and the convolutional LSTM classifier developed by Oktay and Kocer [33] on unseen test data are plotted in Fig. 4, where PD is considered positive and ET, negative.

Based on the interpretation of the ROC as explained in section “Comparison of our method with existing methods on subclinical tremor data”, the curves in Fig. 4 clearly show that at any threshold level, the proposed classifier performs better than the method proposed by Oktay and Kocer. The AUC values support this finding, as the proposed classifier achieves 0.960 (indicating good class separability) while the convolutional LSTM achieves only 0.740 (indicating poorer class separability). This means that the proposed classifier is better at differentiating the classes more accurately than the convolutional LSTM method.

The next experiment used both classifier architectures to attempt to classify the subclinical tremors as PD, ET, or normal physiological tremors. This was done by adding a new output class to the last fully connected layer in both architectures and then training the networks to perform a three-class classification.

Table 3 shows the training, validation, and testing accuracies for each fold when using the network to classify normal physiological tremors, subclinical ET, and subclinical PD tremors. There is a larger variance in the training accuracy for each fold compared to the binary classifier trained previously, as the accuracy varies from 77.9 to 91.2%. This can be attributed to the now much larger training set with each fold having 267–270 evenly balanced training samples, with data oversampled from both the PD and ET classes. On average, the networks have a training accuracy of 85.9%, accurately classifying 232 of the 270 training samples. The validation and testing accuracies are consistently higher in this instance as well, indicating that the measures taken to mitigate overfitting have been successful. Each validation set included 39–42 samples, with a mean validation accuracy of 91.8%. On unseen data, the network can classify the tremors with 90–93.3% accuracy.

**Table 3:** Training, validation, and testing statistics for PD, ET and normal tremors classification with proposed three-class classifier

	Fold 1	Fold 2	Fold 3	Fold 4	Fold 5	Mean accuracy
Training accuracy	0.779	0.861	0.881	0.912	0.864	0.859
Validation accuracy	0.919	0.984	0.826	0.969	0.891	0.918
Testing accuracy	0.900	0.900	0.900	0.933	0.900	0.907

The accuracies of the final subclinical tremor classifiers are shown in Table 4. The accuracy of the proposed classifier was 93.3%. However, in this experiment, while the accuracy of the proposed method remained the same, the performance of the convolutional LSTM reduced significantly. Our method was more than two times more accurate than the convolutional LSTM classifier [33] for the three-class classification problem.

**Table 4:** Accuracy of three-class classifiers on test data

Classifier accuracy	
Proposed classifier	0.933
Convolutional LSTM [33]	0.433

For detecting ET and PD tremors, the proposed classifier demonstrates excellent performance (Table 5) with perfect precision, specificity, and zero FPR, alongside high sensitivity (0.900) and F1 score (0.947). The convolutional LSTM, in contrast, shows only moderate performance with lower values across all metrics. When classifying normal cases, the proposed classifier achieves perfect sensitivity and NPV but with slightly lower precision (0.833) compared to PD and ET cases. This indicates a small yet significant (10% FPR) chance of misclassifying PD or ET cases as normal, which could be concerning in clinical settings. However, the convolutional LSTM performs even worse overall, with its lowest sensitivity (0.400) in detecting normal and ET classes.

**Table 5:** Performance metrics for three-class classifiers on test data

	Sensitivity	Precision	Specificity	F1 Score	FPR	NPV	AUC
PD as the positive class							
Proposed classifier	0.900	1.000	1.000	0.947	0.000	0.952	0.865
Convolutional LSTM [33]	0.500	0.417	0.650	0.455	0.350	0.722	0.555
ET as the positive class							
Proposed classifier	0.900	1.000	1.000	0.947	0.000	0.952	0.950
Convolutional LSTM [33]	0.400	0.500	0.800	0.444	0.200	0.727	0.630
Normal as the positive class							
Proposed classifier	1.000	0.833	0.900	0.909	0.100	1.000	0.950
Convolutional LSTM [33]	0.400	0.400	0.700	0.400	0.300	0.700	0.625

**Fig. 5:**
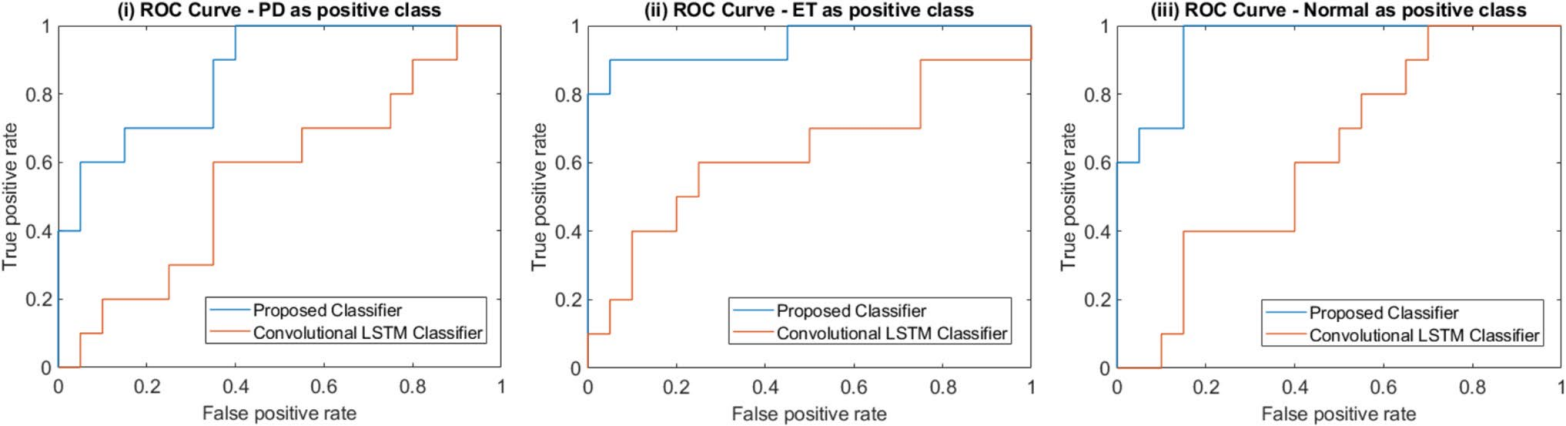
ROC curves for the three-class classifier with **i** PD as the positive class, **ii** ET as the positive class, **iii** Normal as the positive class

The metric-by-metric analysis further highlights the superiority of the proposed classifier. It maintains high sensitivity (0.900–1.000), precision (0.833–1.000), and specificity (0.900–1.000) across all classes. The performance of the convolutional LSTM is consistently lower and displays more variance, missing about half of all positive diagnoses. The proposed classifier also shows a better balance between precision and recall (F1 scores: 0.909–0.947) and a superior discriminative ability across all thresholds (AUC: 0.865–0.950) compared to the convolutional LSTM (F1: 0.400–0.455, AUC: 0.555–0.630). This can also be clearly seen from the ROC curves shown in Fig. 5.

Since it is a three-class classification problem, the ROC curves and performance metrics were calculated by considering three one-vs.-all class metric calculations. The ROC curves are plotted in Fig. 5. The first considered tremors classified as PD as the positive condition and both ET and normal tremor as negative; the second considered ET as the positive case and the other two classes as negative; and finally, the metrics of pathological tremor vs. physiological tremor (normal) were calculated, with the normal tremor as the positive class. From the ROC curves, it is clear that the performance of the proposed classifier is better than that of the convolutional LSTM method since the ROC curves of the proposed method are closer to the top left corner. This is also reflected in the higher AUC values across the board for the proposed method.

The characteristics of the subjects recruited in the study are presented in Table 6 for the interpretation of the generalizability of the classification results. The PD patients recruited are of Hoehn and Yahr stages I to IV, with 35% of stage I, 29% of stage II, 26% of stage III, and 10% of stage IV. The average ages are 70 for the PD patients, 56 for the normal subjects, and 67 for the ET patients, with standard deviations of 7.8, 11, and 9.5 respectively. Males constitute 66.7% of PD patients, while 51.7% of normal subjects and 40.0% of ET patients are male. An estimated medication wear-off period of three hours was used as a reference to characterize the patients. A majority of the PD (68.7%) and ET (86.7%) patients either took no medication or took medication over 3 h before being subject to tremor measurement. The measured upper limbs are the ones reported by the patients to be more tremulous or if there is no obvious difference, the more dominant upper limb is measured. All the results should be interpreted according to the tested conditions.

**Table 6:** Clinical characteristics of the subjects

	PD	Normal	ET
	Resting (n = 51)	Resting (n = 58)	Resting (n = 15)
Mean age, year (SD; range)	70 (7.8; 52–86)	56 (11; 40–80)	67 (9.5; 46–82)
Male, number (%)	34 (66.7)	30 (51.7)	6 (40.0)
Duration since last medication intake, number of patients (%)			
< 3 h	12 (23.5)	–	2 (13.3)
≥ 3 h	34 (66.7)	–	9 (60.0)
Unknown	4 (7.8)	–	4 (26.7)
No medication intake, number of patients (%)	1 (2.0)	–	–

## Discussion

To date, several studies have used machine learning to differentiate between tremor conditions. They vary in the types of models they use and the features utilized to accomplish this classification. The work by Xing et al. [9] showed that random forest classifiers and XGBoost could achieve an accuracy of 84% and 85%, respectively, when used on a combination of features (acceleration measurements and sEMG measurements while holding different postures, including resting, stretching, winging, and vertically winging). A study carried out by Balachandar et al. [10] used sensors in smartphones combined with machine learning models to successfully classify Parkinsonian tremors with a specificity of 97% and, sensitivity of 84% and an impressive 88% total accuracy when classifying ET. The work by de Araújo et al. [11] compared the performance of several different machine learning techniques with different numbers of features to assess Parkinsonian tremors. The best performers were KNNs, followed by logistic regression and random forest algorithms, all of which had accuracy levels above 90%. It was also learned that an increase in the number of features led to an increase in accuracy up to a threshold of 136. Oung et al. [13] were able to distinguish PD from non-PD subjects using KNNs, probabilistic neural networks, and extreme learning machines with classification accuracies of around 90% using empirical wavelet transforms of motion data. NeurDNet [14] is a CNN-based deep learning methodology developed to differentiate between PD and ET, and it displays an impressive 95.5% classification accuracy. Our method shows comparable performance with NeurDNet [14] and outperforms the work of the rest of the above-mentioned work [9, 10, 12] when classifying tremors. However, this may not be a fair comparison as we are classifying among low-amplitude subclinical tremors exclusively.

To better contrast our results with previous work, we selected a study that was similar to our method, using deep learning on accelerometer data, used to classify visible rest tremors due to PD and ET with an accuracy of 85% presented by Oktay and Kocer [33], and trained the same model on our subclinical tremor dataset. The model managed to achieve a classification accuracy of 43.3–65.0% when differentiating subclinical PD and ET tremors, respectively. When compared against this, the proposed method performed better when classifying low amplitude tremors, with an improvement of 30% when classifying between PD and ET over the scheme by Oktay and Kocer [33], and 50% when classifying between PD, ET, and physiological tremors. This could be attributed to the bespoke feature selection process that was applied and the additional analyses and hyperparameter tuning that was performed to optimise the network architecture.

It can therefore be deduced that architectures that have previously been used for tremor classification cannot directly be transferred over and used to classify subclinical tremors, as their defining characteristics may differ. The method proposed by Oktay and Kocer [33] was the most suitable algorithm for comparison because it is one of the most recent AI-based methodologies for tremor classification, and no subclinical tremor classification methodologies have been published. It is important to note that during our study, we did not perform any hyperparameter tuning for the network architecture proposed by Oktay and Kocer [33], and instead used the exact hyperparameters detailed in their paper for training the network to classify subclinical tremors. However, due to the intrinsic differences between the characteristics of subclinical and visible tremor, it may be possible that the network could have performed better if the hyperparameters were adjusted to better suit subclinical tremor differentiation.

It should be noted that the Fourier-transformed data that were input into the developed classifier were intrinsically three-dimensional; however, due to the nature of the tremor data and the signal processing techniques used, the frequency and time windows used to quantify the tremors were standardised for all samples, as mentioned in section “Feature selection”. Since the classifier uses each frequency window as a separate input, a set of sequences of magnitudes corresponding to standardised time intervals were fed as sequence inputs to the LSTM network, thereby transferring all the three-dimensional information of the data into the classifier. The multi-dimensional nature of the information processed for the classification may be the reason for its superior performance, even when dealing with subclinical tremors with high levels of apparent similarity.

An important characteristic of the proposed method, when compared to the method proposed by Okay and Kocer [33], is the low computational power required to train the networks and subsequently classify the tremor signals. Since feature extraction was done separately via an STFT algorithm, the training of the actual LSTM network required much less computational time. The convolutional LSTM developed by Oktay and Kocer used a deep learning convolutional network to extract the features. This took a much longer time to train, and required significantly more computing power. Training converged faster for the proposed method than the convolutional LSTM method [33]. On average, for the same number of iterations, the convolutional LSTM network took approximately 12 min to train on a single CPU, while the proposed architecture only took 17 s on the same hardware. The STFT function used to extract the features was executed in less than 4 s for the entire dataset.

The proposed method yields a classification accuracy of 93.3% when differentiating between PD, ET, and physiological tremors, and 95% when classifying subclinical ET and PD tremors. However, several limitations were identified in this study. The tremor data collected was limited, both in quantity and the demographic variety of the clinical subjects. Future work on this topic can utilise additional data collected from a more diverse group of tremor patients. Due to the size of the dataset, especially the small number of ET samples, it is difficult to ensure that the final unseen testing data is representative of the entire demographic. To make sure that the test set is balanced, we took 10 samples from each category to classify and calculate our metrics. The performance of testing this small sample might not be able to accurately illustrate the performance of the network on a larger, more varied population. However, as illustrated in the “Results” section, we have taken steps to ensure generalization and the metrics seem to support this. A larger sample size can allow for more robust training and validation and improve the confidence of the final network architecture.

Furthermore, this study only considered rest tremors when training and validating the neural network. Although rest tremors are well-classified in the machine learning models [9, 12, 33], the inclusion of more postures seems to almost always lead to better model performance [9, 15, 33]. Therefore, tremor data collected from patients engaging in deliberate activities, such as holding an outstretched pose or drinking from a glass of water, can be used in tandem with rest tremors to develop a more robust framework for classifying subclinical tremors. Some important factors to consider with regard to the applicability of the proposed method are the clinical characteristics of the patients with subclinical tremors and whether the performance of the network outweighs that of tremor differentiation between the tremor types via observation. More studies should be carried out to further improve and validate the proposed method; in particular, this framework can be further validated on larger datasets of subclinical tremors and be used as a clinicians’ aid to validate the performance of the model against a doctor’s diagnosis as the gold standard in a medical setting.

Further efforts are needed to make the model more explainable to better understand the weights and biases of the network and its reasoning in decision-making, such as in the previous work that applied layer-wise relevance propagation for explaining tremor classification in general [34]. This can help build trust with clinicians and also identify the discerning features of subclinical pathological tremors. Nevertheless, this work has shown the feasibility of implementing LSTM networks to discern subclinical rest tremors of PD from those of ET and normal subjects. The presented work holds potential to aid clinicians in differentiating subclinical tremors, which are prevalent in a significant number of PD patients, especially in cases where the diagnosis is complicated or the symptoms are indistinguishable.

## Conclusion

In this paper, we have introduced a novel, non-intrusive methodology for classifying subclinical tremors using a dataset consisting of 122 normal tremors, 83 PD tremors, and 30 ET tremors. A classifier was designed and trained, which could classify subclinical tremors as PD or ET with an accuracy of 95%, and was able to differentiate between PD, ET, and normal subclinical tremors with a similar accuracy of 93.3%. We have shown that our method outperforms a more complex methodology used to classify clinical tremors. Our system needs to be further validated in terms of its applicability in order to assist clinicians in differentiating between movement disorders with subclinical tremors.

## Data Availability

The datasets generated during and/or analysed during the current study are available from the corresponding author upon reasonable request.
